# Fluorescence *In Situ* Hybridization for *MDM2* Amplification as a Routine Ancillary Diagnostic Tool for Suspected Well-Differentiated and Dedifferentiated Liposarcomas: Experience at a Tertiary Center

**DOI:** 10.1155/2015/812089

**Published:** 2015-02-25

**Authors:** Khin Thway, Jayson Wang, John Swansbury, Toon Min, Cyril Fisher

**Affiliations:** ^1^Sarcoma Unit, Royal Marsden Hospital, London SW3 6JJ, UK; ^2^Department of Histopathology, Royal Marsden Hospital, London SW3 6JJ, UK; ^3^Clinical Cytogenetics, Royal Marsden Hospital, Sutton, Surrey SM2 5NG, UK

## Abstract

*Background*. The assessment of *MDM2* gene amplification by fluorescence *in situ* hybridization (FISH) has become a routine ancillary tool for diagnosing atypical lipomatous tumor (ALT)/well-differentiated liposarcoma and dedifferentiated liposarcoma (WDL/DDL) in specialist sarcoma units. We describe our experience of its utility at our tertiary institute. *Methods*. All routine histology samples in which *MDM2* amplification was assessed with FISH over a 2-year period were included, and FISH results were correlated with clinical and histologic findings. *Results*. 365 samples from 347 patients had FISH for *MDM2* gene amplification. 170 were positive (i.e., showed *MDM2* gene amplification), 192 were negative, and 3 were technically unsatisfactory. There were 122 histologically benign cases showing a histology:FISH concordance rate of 92.6%, 142 WDL/DDL (concordance 96.5%), and 34 cases histologically equivocal for WDL (concordance 50%). Of 64 spindle cell/pleomorphic neoplasms (in which DDL was a differential diagnosis), 21.9% showed *MDM2* amplification. Of the cases with discrepant histology and FISH, all but 3 had diagnoses amended following FISH results. For discrepancies of benign histology but positive FISH, lesions were on average larger, more frequently in “classical” (intra-abdominal or inguinal) sites for WDL/DDL and more frequently core biopsies. Discrepancies of malignant histology but negative FISH were smaller, less frequently in “classical” sites but again more frequently core biopsies. *Conclusions*. FISH has a high correlation rate with histology for cases with firm histologic diagnoses of lipoma or WDL/DDL. It is a useful ancillary diagnostic tool in histologically equivocal cases, particularly in WDL lacking significant histologic atypia or DDL without corresponding WDL component, especially in larger tumors, those from intra-abdominal or inguinal sites or core biopsies. There is a significant group of well-differentiated adipocytic neoplasms which are difficult to diagnose on morphology alone, in which FISH for *MDM2* amplification is diagnostically contributory.

## 1. Introduction

Adipocytic tumors are the commonest soft tissue neoplasms [1] and form a large group, which includes lipomas and their histological variants and liposarcomas (LPS). Of the latter, atypical lipomatous tumor (ALT)/well-differentiated liposarcoma (collectively referred to here as WDL) and dedifferentiated liposarcoma (DDL) form the largest subgroup and are considered to represent a morphological spectrum of the same disease entity [2, 3]. There is frequent histologic overlap between different subtypes of adipocytic neoplasm, including, importantly, between benign and malignant groups. The diagnosis of WDL depends on the presence of atypia within predominantly mature adipocytes or fibrous septa, but atypia can be focal or subtle, and distinguishing WDL from various benign adipocytic neoplasms, or even from normal fat, can be challenging, especially in the presence of additional factors such as fat necrosis [4, 5]. A further area of diagnostic difficulty is in distinguishing DDL from other soft tissue sarcomas. DDL is morphologically heterogeneous, usually with the appearance of undifferentiated spindle cell or pleomorphic sarcoma and can have heterologous differentiation towards other mesenchymal lineages [6]. Inflammatory DDL may resemble IgG4-associated sclerosing lesions or inflammatory myofibroblastic tumor [7], histologically low grade pattern DDL can mimic fibromatosis or low grade fibromyxoid sarcoma, and some patterns of WDL/DDL resemble pleomorphic or myxoid liposarcomatous subtypes [8, 9]. The accurate diagnosis of adipocytic neoplasms is crucial, as WDL is more prone to local recurrence than benign adipocytic tumors [10, 11] and has the potential to dedifferentiate, especially within the abdomen/retroperitoneum. The ability to diagnose DDL is useful prognostically, as it has a lower tendency to local recurrence and metastasis compared with both other liposarcomas and other morphologically similar sarcomas such as undifferentiated pleomorphic sarcoma (UPS) or leiomyosarcoma [12].

As several soft tissue sarcomas harbor characteristic genetic abnormalities, molecular genetic and molecular cytogenetic analyses are valuable ancillary diagnostic tools [13]. After early studies showing amplification of the chromosomal 12q13-15 region (which includes several genes such as* MDM2* and* CDK4*) in some sarcoma types including liposarcomas [14, 15],* MDM2* gene amplification, in the form of supernumerary ring and/or giant chromosomes, has been shown to be characteristic of WDL and DDL [16–18].* MDM2* amplification is also associated with other sarcomas such as parosteal osteosarcoma and intimal sarcoma [19, 20], so while it is not entirely specific for WDL and DDL, its assessment by FISH has been developed as an adjunctive tool for their diagnosis [21]. We investigated the utility of assessment of* MDM2* amplification by FISH as an ancillary tool for the histological diagnosis of WDL and DDL and in distinguishing these tumors from other neoplasms in their differential diagnosis in routine diagnostic practice.

## 2. Methods

All cases were formalin fixed and paraffin embedded (FFPE) and comprised consecutive specimens from the routine surgical pathology workload that had fluorescence* in situ* hybridization (FISH) performed for* MDM2* amplification over a 2-year period from March 2011 to March 2013. Case numbers were retrieved from the molecular cytogenetics database (J. S.) and matched with the corresponding histopathological specimens from the electronic patient record. These were specimens in which well-differentiated or dedifferentiated liposarcoma was in the differential diagnosis and included (i) lipomatous tumors with atypical histological features for which a confirmatory positive FISH result was sought, (ii) histologically benign adipocytic neoplasms that were recurrent, large (>10 cm), or sited deeply, and (iii) spindle cell or pleomorphic sarcomas in which DDL was suspected or in the differential diagnosis, due to histologic features or anatomic site. Cases comprised both core biopsy and excision specimens of material biopsied or resected at our center and external cases which were sent for review or second opinion. All diagnoses had been previously made from morphology and immunohistochemistry by one or both of two specialist soft tissue pathologists (K. T. and C. F.). The histopathological reports, slides, and clinical histories were reviewed, and comparison was made between initial and final diagnoses. Clinical information included patient's age and sex and the site and size of lesions. For FISH, 2 *μ*m thick FFPE sections were dewaxed overnight at 60°C, treated with hot buffer wash at 80°C (2-3 hrs) and then with proteolytic enzyme treatment at 37°C, and finally washed in distilled water and then an alcohol series before the addition of* MDM2* and chromosome 12 centromere (CEP12) DNA probes (Vysis* MDM2*/CEP 12 FISH Probe Kit, Abbott Laboratories Ltd., UK). Hybridization was performed overnight according to the manufacturer's protocols. Unless the entire tissue was involved, a stained slide was supplied with the area of interest marked, and this area was generally assessed first for FISH signal patterns. A normal result was of two* MDM2* and CEP12 signals. Signal loss, which is commonly found in thin sections, was ignored for the purposes of this study as being nondiagnostic. Occasional cells with an extra signal were also ignored. Cells with gains of roughly equal numbers of up to eight CEP12 and* MDM2* signals were deemed to be clonal with aneuploidy. The usual pattern of amplification was two to four CEP 12 signals with at least six extra* MDM2* signals. As well as being in greater number, the extra signals were usually smaller and were usually clustered. If no clear result was obtained, or if all the nuclei had a normal signal pattern, then the entire tissue section was screened. As far as possible, overlapping tumor nuclei were also excluded from evaluation. Each case was scored independently by two senior clinical cytogeneticists. If their findings did not match, or if they were suspicious of a low level abnormality, a third scientist was called in to provide an opinion. Representative images of the sections were captured using a cooled charged coupled device camera.

## 3. Results

365 FISH tests were performed in 347 patients in the 2-year period. Tests were repeated in 6 patients (3 due to initial technical failure and 3 on different blocks of the same tumor but with different morphologies). 11 patients had subsequent samples retested, while 1 had two separate lipomatous tumors tested (see FISH results).

### 3.1. Patient and Tumor Characteristics (Table 1)

There were 214 males and 133 females (ratio 1.61 : 1), with median age at diagnosis of 59 years (range 12–95 years) and median tumor size 13.5 cm (range 2–109 cm). The commonest tumor sites were intra-abdominal/retroperitoneal (148), lower limb girdle/inguinal region (88), trunk (57), and upper limb/shoulder (35), with smaller numbers in head and neck (22), lower extremities (13), and thoracic cavity (3). 221 specimens were biopsied or excised in house, and 144 were referred from other hospitals. Where known, 174 cases were resection specimens and 82 were biopsies (most commonly needle core biopsies).

### 3.2. Histological Findings

All specimens had a provisional histological diagnosis made at our institute prior to FISH analysis (Table 2). 122 were diagnosed as benign (most commonly lipomas, spindle cell/pleomorphic lipomas, and intramuscular lipomas). Of 209 cases diagnosed as malignant, there were 145 liposarcomas (73 WDL, 69 DDL, 1 myxoid LPS, and 2 pleomorphic LPS) and 64 other soft tissue neoplasms (most commonly UPS/spindle cell sarcomas), of which the majority were at intra-abdominal/retroperitoneal or inguinal sites (*n* = 57) (necessitating the need to exclude DDL), with small numbers in the abdominal wall, thorax/trunk, and leg. For 34 cases a conclusive histological diagnosis could not be made between a benign adipocytic lesion or WDL; in 19/34 a benign diagnosis was favored but WDL could not be excluded (due to occasional atypical cells, tumor site, or the fact that tumor was recurrent) while in 15/34, WDL had been strongly suspected, but a definite diagnosis was not made.

### 3.3. FISH Results

Of 362 technically successful tests, 170 were positive, that is, showing* MDM2* gene amplification, and 192 were negative, that is, not showing amplification. Of the negatives, 136 had the normal two* MDM2* and CEP12 signals while 56 showed abnormal CEP12 and* MDM2* signals (ranging from 1 to 10 copies of both CEP12 and* MDM2*), suggesting gain (or loss, in 2 cases) involving possibly the whole chromosome 12 (Table 2). These comprised a variety of neoplasms (both benign and malignant), including spindle cell and pleomorphic lipoma and UPS. Since there were equal numbers of CEP12 and* MDM2* signals in these cases and the* MDM2* signals were of usual sizes, there were no features to suggest specific amplification of the* MDM2* region. This implied possible aneuploidy involving chromosome 12, or even hyperploidy of all chromosomes in these neoplasms. In 3 patients, tests were repeated due to initial technical failure; all were external review cases, and the failures may have been due to fixation differences in other laboratories. The subsequent repeat samples were successful and were from material excised at our institute. One patient had 2 separate lipomatous neoplasms tested (1 positive for* MDM2* amplification, the other negative). 11 patients had retesting of the same tumor: 3 had 2 separate blocks from the same tumor tested (all 3 giving consistent results in separate blocks) and 8 had retesting on new samples (6 with core biopsies followed soon after by tumor resections and 2 instances of resampling after 2- and 3-year intervals). Of these 8, 4 were initially external review cases and 4 were internal biopsies. All 8 subsequent samples comprised internal material and of these retested samples, 2 tested positive on both first and second samples, 4 were negative in both, and 2 were initially* MDM2* amplification negative, but subsequently positive. These last 2 samples were initially core biopsies (both internal sampling), with subsequent resection specimens.

### 3.4. Correlation of FISH with Histology

Of the 122 lesions histologically diagnosed as benign, 113 showed no* MDM2* amplification (giving a 92.6% concordance rate between histology and FISH), but 9 showed* MDM2* amplification. These included 4 initially diagnosed as lipomas, 2 intramuscular lipomas, 2 pleomorphic lipomas, and 1 of fibroadipose tissue within scar at the site of previous retroperitoneal tumor. All but the last case were at extra-abdominal sites (extremity or trunk). In all 9 cases, the final diagnosis was amended according to the FISH results.

Of the 73 cases with histologic diagnoses of WDL, 71 showed* MDM2* amplification (giving a 97.3% histology:FISH concordance rate). Both of the 2 non-*MDM2* amplified histological WDL (1 from retroperitoneum, 1 from chest wall), as well as 2 amplified cases, showed abnormal CEP12 signals, with 4–6 copies of probe signals seen. For histologic DDL, 66/69 cases showed* MDM2* amplification (95.7% histology:FISH concordance rate), of which 15 showed additional CEP12 signals. The 3 negative cases all showed abnormal CEP12 signals, with 5–8 copies present. The 2 pleomorphic LPS and 1 myxoid LPS tested did not show* MDM2* amplification. Abnormal CEP12 signals were seen in the 2 pleomorphic LPS, but not the myxoid LPS (in keeping with pleomorphic LPS harboring complex karyotypes typical of other pleomorphic sarcomas and myxoid LPS having balanced translocations and not expected to exhibit aneuploidy). The overall concordance rate of histology with FISH for WDL/DDL was 96.5%. Of the 5 WDL/DDL negative for* MDM2* amplification, 4 had final diagnosis revised (2 presumed DDL revised to spindle cell sarcoma not otherwise specified (NOS), and 2 WDL revised to lipomas), while 1 (which was retroperitoneal, with adjacent unequivocal WDL components) had its histologic diagnosis of DDL retained.

Of the 19 cases where a benign diagnosis was favored histologically but WDL was a possibility, 13 were negative for* MDM2* amplification (68.4% concordance, i.e., 31.6% of histologically “possible WDL” were* MDM2* positive), with 1 of these showing abnormal CEP12 signals. The other 6 were positive for* MDM2* amplification, of which 4 had minor equivocal degrees of histologic atypia and 2 were suspected recurrences of WDL which morphologically resembled normal fat without atypia. All were extra-abdominal (from extremities). In all cases, the final diagnosis was amended according to FISH results.

In contrast, of the 15 cases where WDL was histologically favored, that is, histologically “probable WDL” but not conclusive, 1 failed technically, only 4 were positive for* MDM2* amplification (1 from retroperitoneum, 2 thigh, and 1 lower leg) (28.6% concordance), and 10 were negative (1 with abnormal CEP12 signals). 6/10 negative cases were from intra-abdominal (retroperitoneal) sites and 8/10 had their final diagnosis amended to lipoma variants (4 being classed as true retroperitoneal lipomas). However, in 2 retroperitoneal cases, the histologic features were such that the final report stated that WDL could not be excluded, with advice to monitor for recurrences.

Lastly, of the 64 histological soft tissue sarcomas (many of which were retroperitoneal/intra-abdominal and hence for which DDL was in the differential diagnosis) (sites: 30 retroperitoneal, 16 intra-abdominal/mesenteric, 5 intrapelvic, 6 groin or spermatic cord, and 7 in abdominal wall, thorax/trunk, or leg), 14 (21.9%) (including 9/30 retroperitoneal tumors) showed* MDM2* amplification, with 8 also having abnormal* CEP12* signals. 9 of these 14* MDM2* amplified tumors had their final diagnosis revised to DDL (3 showing heterologous differentiation). The diagnosis was not changed in 2 cases (1 rhabdomyosarcoma (RMS) and 1 malignant peripheral nerve sheath tumor in a patient with neurofibromatosis-1), and in the remaining 3 (2 retroperitoneal RMS and 1 solitary fibrous tumor in the thigh), the final conclusions remained equivocal. There were 48 cases without* MDM2* amplification, of which 31 showed abnormal CEP12 signals and 17 had normal* MDM2* and centromeric signals. However, in 2 (both core biopsies), FISH was repeated on subsequent resection specimens and produced positive results. 2 cases failed technically.

### 3.5. Analysis of Discordant Samples

Patient and tumor characteristics in the cases with discordant histology and FISH were compared (Table 3). For this, the 64 spindle cell neoplasms with a differential diagnosis of DDL (largely due to intra-abdominal/inguinal site but in which no conclusive evidence of DDL was present) were excluded, as were the 3 myxoid or pleomorphic LPS. The categories of “definite” and “probable” diagnoses were combined for each of benign and malignant diagnoses (benign adipocytic lesions and WDL/DDL groups). For benign histological diagnoses, 15/141 specimens were unexpectedly positive for* MDM2* amplification by FISH (10.6%). For histological WDL/DDL 15/157 were unexpectedly negative for* MDM2* amplification (9.6%).

For benign lesions, median tumor sizes for concordant and discordant cases were 9 cm and 15 cm, respectively. 31.5% of concordant cases were intra-abdominal or inguinal, compared with 53.3% of discordant. 21.6% of concordant cases and 45.5% of discordant cases were core biopsies. For malignant (WDL/DDL) cases, median tumor sizes were 18 cm and 13.5 cm for concordant and discordant cases. 79.6% of concordant cases and 66.7% of discordant cases were intra-abdominal or inguinal. 29.2% of concordant cases and 38.5% of discordant cases were core biopsies. Cases with discrepant “benign” histology but positive FISH were therefore on average larger, more frequently occurred intra-abdominally or inguinally and were core biopsy specimens. This is in keeping with the increased likelihood of larger neoplasms being malignant of intra-abdominally or inguinally sited neoplasms representing WDL/DDL and of core biopsies causing sampling error and erroneous “benign” histological interpretations. Cases with discrepant “malignant” histology but negative FISH were smaller, occurred less frequently intra-abdominally or inguinally but again occurred more frequently in cores. This is consistent with smaller, non-intra-abdominal/noninguinal tumors being more likely to represent simple lipoma subtypes, but also similarly subject to sampling error or morphologic distortion on core biopsy.

## 4. Discussion

**Figure 1:**
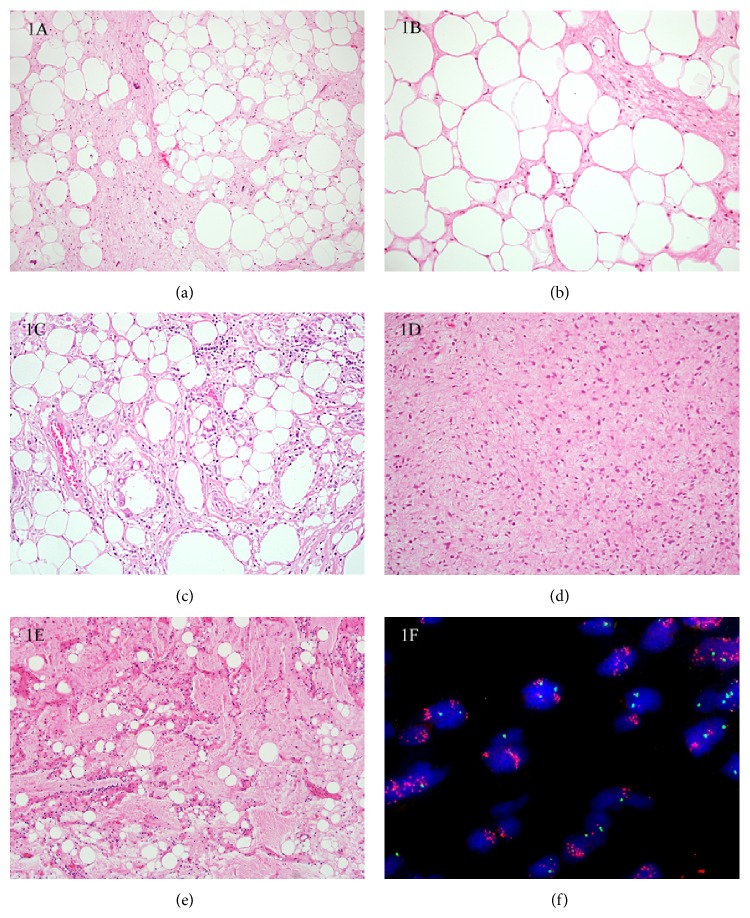
(a) Well-differentiated liposarcoma (WDL). This typical example shows differentiated adipose tissue intersected by thick fibrous septa containing spindle cells with enlarged, hyperchromatic nuclei. (b) This WDL shows lobules of mature adipose tissue, with fibrous septa containing minimal atypia, and can be difficult to distinguish from fibrolipoma or lipoma with fat necrosis. (c) Fat necrosis. This can be extensive, with prominent histiocytes containing plump nuclei, making it difficult to distinguish from WDL. (d) Dedifferentiated liposarcoma (DDL) showing a “low grade” pattern of dedifferentiation can be mistaken for a variety of lesions, including benign neoplasms such as neurofibromas, those of intermediate biologic potential such as fibromatosis, or with other sarcomas such as low grade fibromyxoid sarcoma. FISH for assessment of* MDM2* amplification status is useful in supporting the diagnosis of DDL. (e) This myxoid variant of DDL bears a striking resemblance to myxoid liposarcoma (MLPS). Evidence of* MDM2* amplification with FISH is strongly supportive of DDL, as* MDM2* amplification is not described in MLPS. (f) Fluorescence in situ hybridization for* MDM2* amplification status. The green CEP 12 signals are located on the centromere of chromosome 12 and the red* MDM2* signals are located on the long arm of the same chromosome (12).

The diagnosis of WDL and DDL can be challenging, particularly in core biopsy material where tissue is sparse, or where the histologic features are subtle. Particular areas of confusion include (a) distinguishing WDL (Figures 1(a)-1(b)) from benign mimics (e.g., lipomas including spindle cell/pleomorphic lipomas and fibrolipomas and fat necrosis (Figure 1(c)), (b) distinguishing DDL from other pleomorphic sarcomas in the absence of a well-differentiated component or antecedent history of WDL, and (c) differentiating morphologic variants of WDL/DDL (Figure 1(d)) from other (pleomorphic and myxoid) LPS (Figure 1(e)). These lead to differences in opinion even amongst soft tissue pathologists, and cases sent to tertiary referral centers often include lipomas that are reclassified as WDL and vice versa [4, 5].

Following early work showing* MDM2* amplification in LPS and some MFH [14, 15],* MDM2* amplification has been shown to be characteristic of WDL/DDL [16–18, 22–24] with similar genetic alterations demonstrated between paired well-differentiated and dedifferentiated components [25] (Figure 1(f)). While some earlier studies claimed 100% sensitivity and specificity in distinguishing lipomas from WDL (although also showing that up to 40% of high grade sarcomas harbored* MDM2* amplification) [21], others did not find* MDM2* amplification in all ALT/WDL [16, 26]. Differing results may be due to the use of different techniques in detecting* MDM2* gene amplification, including FISH, real time polymerase chain reaction (RT-PCR), and Southern blotting [16, 26–28], as well as differences in sampling of lesions and different pathologists' morphologic thresholds for making the diagnosis. Most recent studies have utilized FISH, using commercial probes for* MDM2* [21, 29]. While immunohistochemistry for MDM2 has high levels of accuracy, especially when coupled with that for CDK4 and p16 [29–35], the MDM2 antibody can be technically inconsistent [34] and p16 is nonspecific as it is expressed in a variety of nonadipocytic neoplasms. To this end, as FISH is shown to be a robust ancillary molecular cytogenetic technique [36] and its use becomes more widespread routinely; it seems reasonable to use it to assess for* MDM2* amplification in the first instance. Most reported series have used FISH as the diagnostic “gold standard,” with review or reconsideration of the final diagnosis based on its results [37, 38]. However, other studies have based final diagnosis on histologic criteria [29, 39, 40] or a combination of techniques [29].

In this study, we found that where there was a firm histologic diagnosis of benign lipomatous tumor or of WDL/DDL, the concordance rates of histology with* MDM2* amplification results were high (92.6% and 96.5%, resp.). These results are broadly similar to the concordance rates of 85–100% in other published data [37–39, 41, 42], although those studies used cases in which firm histologic diagnoses had been made and did not consider “equivocal” diagnoses. Our series includes a large number of equivocal and uncertain histologic diagnoses, and it should be emphasized that the large majority of specimens analyzed by FISH in this study were those in which there was a level of diagnostic uncertainty; hence unequivocal cases of lipomas or DDL with adjacent WDL component were not tested. The concordance rate of histology and FISH seen here would almost certainly be higher if more diagnostically certain cases had been included. In dividing cases into two “levels” of histological uncertainty, we found that 31.6% of “possible” WDL were* MDM2* amplified, while 28.6% of “probable” WDL were* MDM2* amplified. All cases falling into this equivocal category therefore have a similar rate of positive FISH, and it would therefore seem prudent to perform FISH on any case of possible WDL with an element of uncertainty (irrespective of the degree of perceived histologic atypia).

In the 64 soft tissue neoplasms in which pure DDL was in the differential diagnosis,* MDM2* was amplified in only 21.9%. Most of these cases comprised spindle or pleomorphic sarcomas (not otherwise specified) without specific morphologic or immunohistochemical differentiation, other than scanty single marker expression (e.g., SMA only, or desmin only, or scanty CD34 only), which were not possible to further characterize. Small numbers of these cases were assigned provisional diagnoses, for example, rhabdomyosarcoma, if there was focal expression of appropriate markers (e.g., clear cut desmin with myogenin or MyoD1) (Table 2). The significance of this finding of* MDM2* amplification in 21.9% is uncertain, since up to 40% of other soft tissue sarcomas can harbor amplified* MDM2* [21, 22, 39, 40]. This and the fact that DDL can have a variety of appearances ranging from bland fibromatosis-like to UPS-like and exhibit several different types of heterologous differentiation [6, 7, 43–46] mean that unless there is an antecedent history of WDL or adjacent WDL component, a diagnosis of DDL cannot always be proven, even at sites where it is likely. Coindre et al. have previously shown that most retroperitoneal UPS represent DDL [43], although of our 30/64 sarcomas that were retroperitoneal, only 9 showed* MDM2* amplification, a lower figure than expected. It could be said that, for retroperitoneal sarcomas, FISH for* MDM2* is helpful in supporting a diagnosis of DDL rather than another sarcoma type. For example, of 201 spindle cell tumors studied by Kashima et al., 7 had* MDM2* amplification (3 spindle cell sarcomas NOS, 2 osteosarcomas, and 2 myxofibrosarcomas), of which all were retroperitoneal or intra-abdominal, with some on subsequent review showing WDL components, and these were all reclassified as DDL [41].

*MDM2* amplification status by FISH could be of therapeutic importance, as amplified neoplasms, irrespective of precise histologic subtype, might be amenable in the future to targeted treatment with MDM2 antagonists. True retroperitoneal lipomas are rare but increasingly recognized [47] and show clinicopathologic and genetic features (including lack of* MDM2* amplification) more akin to lipomas than WDL [47] such that their identification by FISH is prognostically useful. Positive FISH for* MDM2* would also be useful in excluding pleomorphic and myxoid LPS (both of which can be mimicked by DDL but are virtually unknown intra-abdominally) [8, 9].

An interesting facet of this study is that despite the high histology:FISH concordance rates for clear-cut histologic lipomas and WDL/DDL (as also shown in previous studies), there is a dramatically lower histology:FISH concordance rate for equivocal cases of differentiated adipocytic neoplasms, despite histologic diagnoses by specialist soft tissue pathologists. This highlights that there exists a subgroup of microscopically equivocal well-differentiated lipomatous neoplasms that elude definitive histological diagnosis. If FISH was taken as gold standard, this questions whether, in specific contexts, prior detailed evaluation of differentiated adipocytic lesions by surgical pathologists might essentially be rendered less crucial than* MDM2* FISH. As with all ancillary tests that accompany histology and taking note of the small rate of both technical failures and what appear to be false negative results, we still recommend that FISH should be interpreted strictly in the context of the histological and clinical findings.

Since FISH is both labor and cost intensive and morphologically clear-cut cases of WDL/DDL do not require ancillary diagnostic confirmation, it is important to determine when it would be most useful and cost efficient for diagnosis. From their studies of trunk and extremity neoplasms, Zhang et al. recommended that lipomatous tumors that are recurrent and large (>15 cm) or show possible cytologic atypia are indications for FISH [37]. Neuville et al. also recommended that all poorly differentiated abdominal or retroperitoneal sarcomas be tested [42]. Le Guellec et al. recently showed similarities in histology, genomic profile, and clinical behavior of patients with peripheral UPS with* MDM2* amplification and peripheral conventional DDL which strongly suggested that peripheral UPS with* MDM2* amplification in fact represents DDL [48].

In this series, we found that, for histologically benign-appearing adipocytic neoplasms,* MDM2* amplification was more frequently found in those that were larger or in “classical” (intra-abdominal or inguinal) sites for WDL and in core biopsy specimens. Likewise, in cases of probable WDL/DDL a negative FISH result was more common in core biopsies, smaller lesions, and those not sited intra-abdominally or inguinally. External review cases that had FISH performed interestingly showed fewer discrepancies between histology (reviewed at our tertiary center) and FISH, but this highlights the robustness of FISH technique on referral material [4].

## 5. Conclusions

Our experience of FISH for testing* MDM2* amplification shows high concordance in established histological diagnoses of lipoma and WDL/DDL. The lower concordance for cases with equivocal histological diagnoses is an issue for debate, highlighting both the merit of using FISH as a diagnostic adjunct for all equivocal well-differentiated adipocytic neoplasms and the fact that there might exist a group of histologically differentiated adipocytic tumors needing more detailed morphologic and molecular characterization. There is particular value in performing FISH in core biopsies, larger adipocytic neoplasms with bland histology, and those occurring in “classical” inguinal or intra-abdominal sites. However, FISH results should not be relied on exclusively, and, as for any other ancillary diagnostic tests, should be interpreted in light of the histological and clinical findings.

## Figures and Tables

**Table 1:** Patient and tumor characteristics.

Patient/tumor characteristics	Total
Male	214 (61.7%)
Female	133 (38.3%)
Median age	59 years (range 12–95 years)
Tumor size (where available from the gross specimen or cross-sectional imaging)	13.5 cm (range 2–109 cm)
Tumor site	
Intra-abdominal	148
Retroperitoneum	(113)
Bowel/mesentery	(25)
Pelvis	(10)
Inguinal/lower limb girdle	88
Thigh	(47)
Spermatic cord	(19)
Groin	(14)
Buttock/perineum	(8)
Trunk	57
Back	(22)
Chest wall	(17)
Abdominal wall	(14)
Breast	(4)
Upper limb/shoulder	35
Shoulder	(18)
Arm	(11)
Axilla	(4)
Hand	(1)
Head and neck	22
Neck	(14)
Mouth/jaw	(5)
Scalp/forehead	(2)
Ear	(1)
Lower extremities	13
Knee	(6)
Calf	(4)
Foot	(3)
Thoracic cavity (pleura, mediastinum, and lung)	3

**Table 2:** Comparison of histological tumor type and FISH results.

Histological diagnosis	Total	MDM+	MDM−	MDM2−, with multiple copies of CEP12 and *MDM2* signals
Benign	122	9	113	20
Lipoma	76	4	72	9
Intramuscular lipoma	12	2	10	0
Spindle cell lipoma	17	0	17	5
Pleomorphic lipoma	9	2	7	5
Fat necrosis	2	0	2	0
Lipoblastoma	2	0	2	0
Lipoleiomyoma	1	0	1	1
Hibernoma	1	0	1	0
Nevus lipomatosis	1	0	1	0
Fibroadipose tissue/scar	2	1	1	0
Liposarcoma	145	137	8	10
WDL	73	71	2	5
DDL	69	66	3	3
Myxoid LPS	2	0	2	0
Pleomorphic LPS	1	0	1	2
Equivocal cases	34 (including 1 technical fail)	10	23	0
Possible WDL/DDL	19	6	13	0
Probable/suspected WDL/DDL	15	4	10	0
Other soft tissue sarcomas/malignancies	64 (including 2 technical fails)	14	48	26
Undifferentiated pleomorphic sarcoma	28	5	21	14
Spindle cell sarcoma (NOS)	19	2	17	8
Rhabdomyosarcoma	5	3	2	2
Solitary fibrous tumor	3	1	2	1
Leiomyosarcoma	3	1	2	1
Malignant peripheral nerve sheath tumor	2	1	1	0
Osteosarcoma	1	1	0	0
Inflammatory myofibroblastic tumor	1	0	1	0
Poorly differentiated carcinoma	2	0	2	0

**Table 3:** Comparison of patient and tumor characteristics in cases with concordance or discrepancies of histology and FISH.

Histological diagnosis	MDM2 amplified	Total	Sex of patients	Median age (years)	Median size of tumor (cm)	Classical sites for WDL/DDL (intra-abdominal or inguinal)	Review cases	Core biopsies	Resection specimens
Benign (definite/provisional)	−	126	84 M : 43 F	50.5	9	40 (31.5%)	60 (47.2%)	16 (21.6%)	58 (78.4%)
	+ (i.e., discrepant with histology)	15	8 M : 7 F	59	15	8 (53.3%)	5 (33.3%)	5 (45.5%)	6 (54.5%)

Liposarcoma (definite/provisional)	− (i.e., discrepant with histology)	15	5 M : 12 F	64	13.5	10 (66.7%)	2 (13.3%)	5 (38.5%)	8 (61.5%)
	+	142	92 M : 50 F	63	18	113 (79.6%)	27 (19.0%)	31 (29.2%)	75 (70.8%)
